# The fate of assimilated carbon during drought: impacts on respiration in Amazon rainforests

**DOI:** 10.1098/rstb.2007.0021

**Published:** 2008-02-11

**Authors:** P Meir, D.B Metcalfe, A.C.L Costa, R.A Fisher

**Affiliations:** 1 School of Geosciences, University of EdinburghEdinburgh EH8 9XP, UK; 2 Department of Forest Ecology and Management, Swedish University of Agricultural SciencesUmeå 901-83, Sweden; 3 Departamento de Meteorologia, Centro de Geociências, Universidade Federal do ParáRua Augusto Correa, n° 01, CEP 66075-110 Belém, Pará, Brazil; 4 Department of Animal and Plant Sciences, University of SheffieldSheffield S10 2TN, UK

**Keywords:** net ecosystem exchange, drought, respiration, leaf, woody tissue, soil

## Abstract

Interannual variations in CO_2_ exchange across Amazonia, as deduced from atmospheric inversions, correlate with El Niño occurrence. They are thought to result from changes in net ecosystem exchange and fire incidence that are both related to drought intensity. Alterations to net ecosystem production (NEP) are caused by changes in gross primary production (GPP) and ecosystem respiration (*R*_eco_). Here, we analyse observations of the components of *R*_eco_ (leaves, live and dead woody tissue, and soil) to provide first estimates of changes in *R*_eco_ during short-term (seasonal to interannual) moisture limitation. Although photosynthesis declines if moisture availability is limiting, leaf dark respiration is generally maintained, potentially acclimating upwards in the longer term. If leaf area is lost, then short-term canopy-scale respiratory effluxes from wood and leaves are likely to decline. Using a moderate short-term drying scenario where soil moisture limitation leads to a loss of 0.5 m^2^ m^−2^ yr^−1^ in leaf area index, we estimate a reduction in respiratory CO_2_ efflux from leaves and live woody tissue of 1.0 (±0.4) t C ha^−1^ yr^−1^. Necromass decomposition declines during drought, but mortality increases; the median mortality increase following a strong El Niño is 1.1% (*n*=46 tropical rainforest plots) and yields an estimated net short-term increase in necromass CO_2_ efflux of 0.13–0.18 t C ha^−1^ yr^−1^. Soil respiration is strongly sensitive to moisture limitation over the short term, but not to associated temperature increases. This effect is underestimated in many models but can lead to estimated reductions in CO_2_ efflux of 2.0 (±0.5) t C ha^−1^ yr^−1^. Thus, the majority of short-term respiratory responses to drought point to a decline in *R*_eco_, an outcome that contradicts recent regional-scale modelling of NEP. NEP varies with both GPP and *R*_eco_ but robust moisture response functions are clearly needed to improve quantification of the role of *R*_eco_ in influencing regional-scale CO_2_ emissions from Amazonia.

## 1. Introduction

Respiration returns carbon dioxide (CO_2_) to the atmosphere that was originally removed from it by photosynthesis. CO_2_ is also released to the atmosphere episodically in single oxidation events, most notably fire. In combination, these processes govern the short-term carbon economy of a forested region. However, while terrestrial photosynthesis is performed by one main organ, the leaf, respiration occurs in all living cells and this simple distinction demands consideration of the metabolism of a mosaic of ecosystem components.

Atmospheric inversion studies have provided indirect evidence that Amazonia responds to climatic perturbation at large scale: the interannual flux anomaly is globally significant for tropical South America, showing CO_2_ emissions of up to 1.5 Pg C yr^−1^ during some years, and net CO_2_ acquisition during others (Rodenbeck *et al.* 2003; Zeng *et al.* 2005). Large net emissions have been associated with strong El Niño events impacting Amazonia (Zeng *et al.* 2005). Ecosystem modelling analyses are capable of approximately replicating this interannual pattern in regional-scale CO_2_ fluxes (Tian *et al.* 1998; Zeng *et al.* 2005) and have indicated that El Niño drought-related reductions in net ecosystem production (NEP) with respect to non-El Niño years can be as large as 1 Pg C yr^−1^ or more, owing to declines in photosynthesis coupled with increases in respiration. However, fire incidence also increases under drought stress and thus contributes to the observed El Niño-correlated emission anomalies. Fire-sourced CO_2_ emissions during drought have been difficult to quantify with high precision, but the available data indicate that they have the potential to be at least as large as the modelled declines in NEP (van der Werf *et al.* 2004). The size of the modelled change in regional NEP is also uncertain: although a reduction in gross primary production (GPP) is expected under moisture limitation, there is little evidence to support the short-term drought-correlated increases in ecosystem respiration reported in modelling studies and some evidence to the contrary (e.g. Saleska *et al.* 2003).

Good estimates of NEP require accurate representation of both photosynthesis and respiration, but an historical bias in research towards the former has left respiration less well parametrized, with consequent uncertainty in our understanding of the feedbacks resulting from land–atmosphere CO_2_ exchange across Amazonia. If we are to understand climatic influences on the Amazon carbon balance over the coming decades, then we must first be confident of how alterations in NEP take place at short time scales such as seasonally, or interannually, under episodic climate perturbations like El Niño. NEP represents the difference between carbon that is acquired and respired by an ecosystem (equation (1.1)). Our analysis focuses on using field data from Amazon rainforests as the basis for estimating respiration in each component, and how it changes under moisture limitation, particularly at the interannual time scale,

(1.1) $$\text{NEP}=\text{GPP}-R_{\text{eco}}=\text{GPP}-(R_{\text{l}}+R_{\text{w}}+R_{\text{s}}),$$

where *R*_l_, *R*_w_ and *R*_s_ represent the aggregate component flux contributions to *R*_eco_ from leaves, woody tissue (live and dead) and soil, respectively.

## 2. Climatic influences on respiration

What are the magnitudes of the relevant climatic drivers? Although there is variability among predictions of twenty-first century climatic change for Amazonia, most climate models predict some kind of drying and warming, with the strongest drying scenario indicating a 65% reduction in precipitation (Cubasch *et al.* 2001). Long-term climate data from the twentieth century are much less extreme: they show non-significant changes in precipitation allied with slight warming, although these overall patterns mask important regional and interannual differences (Malhi & Wright 2004). Focusing on the shorter-term climatic impacts of the principal interannual climate perturbation of El Niño, during the 1997/1998 event, precipitation reductions were large, especially for the eastern Amazon, reaching 50% of the long-term mean (Uppala *et al.* 2005). Maximum temperature anomalies were larger during the daytime (average anomaly of maximum=+1.7°C) than the night-time (average anomaly of minimum=+0.5°C), with an average increase in solar radiation of 30.7 W m^−2^ and concomitant decreases in atmospheric humidity yielding a mean midday water vapour pressure deficit anomaly of +0.4 kPa (Uppala *et al.* 2005). The effects of these climatic anomalies on NEP are surprisingly poorly understood. For example, observed changes to NEP in European forests during widespread drought in 2003 were, on average, non-significant (Reichstein *et al.* 2007) because declines in photosynthesis were partially balanced by declines in *R*_eco_. These measured temperate zone declines in respiration during drought contradict recent modelling of the same processes in Amazonia, and thus further prompt the need to consider the response to drought by *R*_eco_ in tropical rainforest ecosystems using direct observations.

## 3. Leaves

Leaf respiration (*R*_l_) comprises 10–40% of the respiratory emissions of CO_2_ to the atmosphere by terrestrial ecosystems (Wright *et al.* 2006). In ecosystems where leaf area is large and temperatures high, the contribution to the carbon budget from foliar respiration can be significant. Although respiration occurs during both day and night, measurement is usually made on leaves in the absence of light so that the respiratory flux of CO_2_ during the dark can be distinguished from that released during photosynthesis, which itself varies with the photosynthetic process, often declining strongly with irradiance (Brooks & Farquhar 1985). *R*_l_ during the night ranges between 2.0 and 7.0 t C ha^−1^ yr^−1^ on a ground area basis in tropical rainforests (Meir 1996; Malhi *et al.* 1999; Chambers *et al.* 2004; R. Vale 2002, unpublished data).

Respiration in leaves yields energy used for the maintenance of leaf metabolism and structure in mature leaves, and for the construction of new leaves. Maintenance respiration is the larger component, is temperature sensitive (Atkin *et al.* 2005), and correlates positively with irradiance, with the tissue concentration of nutrients, such as nitrogen or phosphorus, and thus also with photosynthetic capacity (Meir *et al.* 2001; Wright *et al.* 2006). The impact of moisture limitation on leaf respiration has received less attention. Stomatal closure in response to drought can lead to higher leaf temperatures, and lower photosynthetic rates, but the reported effects on maintenance respiration in leaves differ. Laboratory experiments have mostly suggested that the net effect is small (Flexas *et al.* 2006). For example, at a level of moisture limitation sufficient to cause a general failure of photochemistry and biochemistry, instead of declining, leaf respiration rates were unaltered while electron flow to the alternative oxidative pathway increased from 10 to 40%, with a concomitant decrease by 32% in electron flow to the mitochondrial cytochrome *c* pathway (Ribas-Carbo *et al.* 2005). By contrast, in field measurements on 208 species from sites varying widely in rainfall, Wright *et al.* (2006) showed woody plants in dry environments to have higher leaf respiration rates, and indicated an average 50% increase in leaf respiration for a rainfall reduction from 2000 to 500 mm yr^−1^. These higher leaf respiration rates possibly reflect adaptation, or longer-term acclimation, to drier conditions where, although stomatal closure may constrain photosynthesis, more respiration occurs owing to an increased need to maintain high vacuolar concentrations of osmotically active solutes (Wright *et al.* 2006), or a need to avoid the accumulation of cellular redox equivalents and free oxygen radicals resulting from decreased photosynthesis and increased photoinhibition (Macherel & Atkin in review). The general physiological response to drought by *R*_l_ is thus unclear, although initial experimental data from an eastern Amazon site suggest increases in respiration rate on a leaf area basis after extended artificial soil moisture limitation of 4–5 years (table 1), and this is consistent with dry season measurements at a transition forest in Sinop (Miranda *et al.* 2005).

To fully account for changes in leaf respiration on a ground area basis, the effect of alterations in canopy structure must also be considered. Moisture limitation can cause a decline in leaf area index (LAI) in dry or dry–wet transition zones between savannah and rainforest (Pennington *et al.* 2000). Although seasonal LAI measurements are scarce in tropical rainforests (Carswell *et al.* 2002), two recent artificial droughting experiments in the eastern Amazon (at Tapajos and Caxiuana) have shown LAI reductions of 20% or more (approx. 1 m^2^ m^−2^ over 2 or more years) after reducing rainfall input by approximately 50% (Nepstad *et al.* 2004; Fisher *et al.* 2007). The effect of a loss in LAI is linear in terms of the change in respiration caused by a reduction in leaf biosynthesis, although alterations to leaf longevity and the decline in maintenance respiration with height in the canopy (Domingues *et al.* 2005) could modify this linearity. The metabolic cost of biosynthesis is approximately 0.25(±0.02) g C respired per gram of biomass constructed (Penning de Vries 1975). Therefore, assuming mean leaf maintenance respiration at 25°C to be 0.6(±0.2) μmol C m^−2^ s^−1^ (Chambers *et al.* 2004; Domingues *et al.* 2005), leaf longevity to be 12 months, and an average leaf mass per unit area of 100 g m^−2^, a loss in LAI of 0.5 m^2^ m^−2^ in 1 year would accrue a reduction in construction respiration of 0.07(±0.01) t C ha^−1^ yr^−1^ and a reduction in maintenance respiration of 0.6(±0.2) t C ha^−1^ yr^−1^. Combined with the effect of a 0.5°C night-time warming anomaly (1997/1998 El Niño; Uppala *et al.* 2005), this yields an overall reduction in *R*_l_ of 0.5(±0.2) t C ha^−1^ yr^−1^. Such short-term alterations in canopy-scale leaf respiration are similar in magnitude to the (annual) NEP reported for some Amazonian rainforests (Ometto *et al.* 2005), although this only accounts for changes during darkness; changes in daytime leaf respiration have not been estimated (i.e. assumed=0) given the lack of available data on drought responses in this variable. Over the longer term, sustained moisture limitation (greater than 2 years) could lead to different effects of *R*_l_ on NEP: leaves formed entirely under conditions of significant moisture limitation may be altered in structure (e.g. increased mass per unit area), with consequent increases in leaf respiration on a leaf area basis and, potentially, a canopy basis (cf. table 1)

## 4. Woody tissue

Emissions of CO_2_ from woody tissue in forest ecosystems (*R*_w_) are divided into two main components: the flux from living woody tissue (*R*_wc_) and the flux from decomposing coarse necromass (*R*_wn_). Fluxes from *R*_wc_ are thought to comprise up to 18% of GPP, or 5.1 t C ha^−1^ yr^−1^ (Cavaleri *et al.* 2006). Those from *R*_wn_ vary strongly with precipitation and contain uncertainty of up to 50% (Rice *et al.* 2004). Where detailed measurements have been made, annualized mass loss rates in tropical rainforests suggest *R*_wn_ emissions of 0.5–1.9 t C ha^−1^ yr^−1^ (Chambers *et al.* 2004; Rice *et al.* 2004; Hutyra *et al.* 2007).

The CO_2_ comprising the flux from living woody tissue has two origins, the woody tissue itself and the soil. Mitochondria in living parenchymatic, cambium and phloem cells in wood respire and release CO_2_ into the surrounding fluid, where it dissolves and dissociates according to pH and temperature, following Henry's law. Part of this CO_2_ ultimately diffuses radially and evolves into the atmosphere, but part of it is transported away from the locus of production dissolved in water columns in the xylem, to evolve elsewhere in the canopy or to be refixed by photosynthesis. However, the xylem stream originates in the soil where CO_2_ respired by both roots and microbes dissolves to saturation point, creating an additional and strong CO_2_ source within woody tissue. Hence, the CO_2_ that is released from bark is a product of a mixture of autotrophic and heterotrophic respiration, is dependent on the diffusion properties of live and dead cells in wood, and is sensitive to rates of sap flux (Levy *et al.* 1999; Bowman *et al*. 2005). Different organ-scale metrics by which canopy-scale live woody tissue CO_2_ effluxes (*R*_wc_) may be calculated have been suggested (Ryan 1990; Levy & Jarvis 1998). More recently, Cavaleri *et al.* (2006) made measurements throughout the vertical profile of a Costa Rican rainforest canopy and demonstrated that small diameter branches and lianas high in the canopy were significant sources of CO_2_, especially during the day, the period when CO_2_ evolves from the transpiration stream, diffusing rapidly to the atmosphere through the thinnest sections of tissue and bark.

One solution to quantify *R*_wc_ is to measure changes in emissions caused individually through alterations in maintenance and growth respiration in all above-ground woody tissue elements, and in the transport of heterotrophic and autotrophic CO_2_ by the xylem stream. An alternative and functionally appropriate method is to use the strong observed relationship between *R*_wc_ and LAI for forest ecosystems (figure 1). *R*_wc_ probably scales with LAI for reasons of economy in respiration and transpiration (Shinozaki 1964; Meir & Grace 2002), and this relationship should thus hold functionally as well as biogeographically. Since LAI varies with strong moisture limitation, the *R*_wc_–LAI relationship can be used to quantify the change in CO_2_ efflux from live woody tissue associated with short-term drought. Applying the same reduction in LAI (0.5 m^2^ m^−2^ yr^−1^) used to estimate changes in *R*_l_, figure 1 predicts a reduction in *R*_wc_ of 0.5(±0.1) t C ha^−1^ yr^−1^. These estimates suggest that the impact of drought on *R*_wc_ is similar in direction and magnitude to changes in *R*_l_.

The breakdown of necromass leading to *R*_wn_ is estimated as a function of the rate of loss of dry mass over time, described by a decay constant, *k* (yr^−1^), where *k* for coarse necromass varies among studies between 0.1 and 0.3 yr^−1^ (mean=0.197 (±0.04) yr^−1^, *n*=6; Kira 1976; Yoneda 1990; Chambers *et al.* 2000; Eaton & Lawrence 2006; Palace *et al.* 2007). Irrigation experiments have demonstrated that *k* declines strongly under moisture limitation by a factor of 2.4 or more (Vasconcelos *et al.* 2007), and despite variation in tissue density, moisture also dominates the rate of respiration in decomposing wood, with up to fivefold differences in CO_2_ efflux rates for woody tissue moisture contents varying between 0.5 and 1.5 g H_2_O g^−1^ dry woody biomass (Chambers *et al.* 2000; Keller *et al.* 2004). Accounting for natural spatial variation in necromass moisture content and consequent decomposition rates, Rice *et al.* (2004) proposed a stand-scale value for *k* of 0.113 yr^−1^ for an eastern Amazon forest at Tapajos.

Although drought reduces the rate of decomposition, where sufficient moisture limitation increases tree mortality, then the net effect on *R*_wn_ is positive. Mortality measurements are dependent on the death of only a few trees per 1 ha plot, so the use of data from multiple plots is essential for estimating the overall effects of drought on *R*_wn_. Mortality impacts following short-term drought caused by strong El Niño events vary for tropical rainforests globally, with a median increase of 1.2% for observational studies both in Amazonia and globally (Meir & Grace 2005; *n*=46 plots). Mean standing biomass varies substantially across the Amazon basin (Baker *et al.* 2004) and assuming an increase in mortality of 1.1%, a stand scale *k* of 0.113 (Rice *et al.* 2004) and the lower estimate of 2.4 for the factor by which *k* declines under drought (Chambers *et al.* 2001; Vasconcelos *et al.* 2007), the estimated maximum effect on *R*_wn_ is an increase of 0.13–0.18 t C ha^−1^ yr^−1^, although up to 25% of this flux may instead join the soil organic matter pool (Chambers *et al.* 2001). Thus, the short-term impact of drought on respiration in necromass is likely to marginally increase *R*_eco_ during a drought, although this small increase in *R*_wn_ may be followed by larger fluxes under subsequent increased rainfall, reflecting the increase in the necromass carbon pool under the original period of drought.

## 5. Soil

The efflux of CO_2_ from the soil (*R*_s_) is the largest component of the respiration budget, ranging in size from approximately 11 to 22 t C ha^−1^ yr^−1^, so small fractional changes in this component can influence NEP significantly. *R*_s_ comprises respiration by plant roots (autotrophic respiration, *R*_sa_) and microbes (heterotrophic respiration, *R*_sh_). Recent focus (Trumbore 2006; Metcalfe *et al*. 2007) has emphasized the need to separate *R*_sa_ and *R*_sh_ to improve the mechanistic understanding of *R*_s_. Evidence at seasonal and interannual time scales suggests that the effects of variation in moisture availability on *R*_s_ in tropical rainforests are much larger than temperature. Using chamber measurements, Davidson *et al.* (2000) demonstrated declines of more than 50% in *R*_s_ with changes in soil matric potential of −0.001 to −10 MPa. Subsequent studies have made similar findings (e.g. Schwendenmann *et al.* 2003; Sotta *et al.* 2004, 2007), although conflicting evidence of moisture limitation on *R*_s_ has been reported in one case (Saleska *et al.* 2003; Davidson *et al.* 2004). Using eddy covariance measurements of net ecosystem exchange from a seasonally dry forest at Sinop in Mato Grosso, Vourlitis *et al.* (2005) showed that ecosystem respiration, probably dominated by *R*_s_, declined more strongly in response to moisture constraints than did maximum photosynthesis (figure 2). This moisture sensitivity in net ecosystem exchange is not restricted to climatically marginal sites: eddy covariance data from an eastern Amazon site at Tapajos on deeply weathered oxisols have also been used to show how dry season declines in *R*_s_ contribute to increased net CO_2_ assimilation rates when compared with wet season values (Saleska *et al.* 2003; Hutyra *et al.* 2007).

A fully mechanistic analysis of the impact of moisture limitation on respiration in soil is complicated by the need to quantify both *R*_sh_ and *R*_sa_ in the context of vertical differences in soil moisture along the soil profile, of differences in the supply of respiratory substrate and owing to the as yet largely unquantified role of mycorrhizae (Davidson *et al*. 2006; Meir *et al.* 2006). As a consequence, *R*_s_ is often still represented as an empirical function of environmental constraints. Large-scale modelling studies of Amazonian NEP during El Niño-caused drought have tended to emphasize the enhancement of *R*_sh_ under marginal associated warming (Zeng *et al.* 2005) even though any theoretical temperature response by *R*_s_ is dominated in field data by the availability of moisture (Sotta *et al.* 2007). The response to moisture has been found to be a linear (Davidson *et al.* 2000) or parabolic (Schwendenmann *et al.* 2003; Sotta *et al.* 2007) function of soil water potential, although mixtures of weak and strong responses in *R*_s_ to experimental changes in soil moisture have also been noted (Vasconcelos *et al.* 2004). Recent multi-scale field data are thus establishing the importance of low soil moisture as the dominant short-term climatic constraint on *R*_s_, and indicate that moisture limitations can cause reductions in *R*_s_ of 10–30%, potentially equating to 2.0(±0.5) t C ha^−1^ y^−1^ or more (e.g. Saleska *et al.* 2003; Vourlitis *et al.* 2005; Sotta *et al.* 2007), notwithstanding the potential for efflux pulses following dry period rainfall events (Hutyra *et al.* 2007). Overall, field evidence shows that if strong short-term moisture limitation is experienced, for example, during El Niño-caused drought, it is very likely to cause significant reductions in *R*_s_. The key now will be to specify more accurately the *R*_s_-soil moisture response across the Amazon basin as part of a mechanistically and spatially consistent model of *R*_s_.

## 6. Conclusions

Process modelling of NEP has historically been informed by more data on photosynthesis than on respiration. Although new flux measurements at the ecosystem scale are emerging, improved insight into the component respiratory processes is necessary to properly test and specify model outcomes because these separate processes respond to environmental changes in different and nonlinear ways, sometimes also interacting. Appropriate field data remain relatively sparse, but this analysis shows that the short-term drought responses of the component processes of *R*_eco_ are larger than, or show notable deviations from, previous estimates.

The clearest of the impacts of drought on *R*_eco_ takes place in the soil and this has been observed at seasonal and 1–2 year time scales. Part of the drought-related decline in *R*_s_ results from the decline in GPP. Additionally, some carbon that is not respired owing to moisture limitations on *R*_sh_ is probably retained temporarily in the soil as labile organic material. Improved modelling of the impact of these processes on *R*_s_ will require better understanding of the interactions between *R*_s_ and GPP, and of the effects of moisture limitation on water and substrate supply to respiring cells in soils that differ hydraulically across Amazonia.

The separate impacts of short-term drought on respiration in woody and leafy tissue are directly altered by structural change in leaf area and mortality. Leaf respiration and CO_2_ effluxes from live woody tissue will both decline over the short term if leaf area is lost during drought, although physiological acclimation to drought may modulate the impact on *R*_eco_ over the longer term. The total emissions of CO_2_ from necromass increase when mortality increases, but the main impact on emissions is held in check until the moisture content of the decomposing tissue rises.

To summarize drought-related alterations to *R*_eco_, in the 1- to 2-year drought scenario used for our calculations above, we estimate a loss in LAI of 0.5 m^2^ m^−2^ y^−1^; declines in *R*_l_, *R*_wc_ and *R*_s_ of 0.5(±0.2), 0.5(±0.1) and 2.0(±0.5) t C ha^−1^ y^−1^, respectively; and an increase in *R*_wn_ of 0.13–0.18 t C ha^−1^ yr^−1^. Drought is thus very likely to cause an overall decline in *R*_eco_ of tropical rainforests over seasonal or interannual time scales, with differing effects on each component of *R*_eco_. The largest component, *R*_s_, is especially sensitive to the water-holding characteristics of soil, and understanding this variation over biogeographic space is a research priority: GPP and *R*_s_ are both affected if water is unavailable to plants.

In the specific case of El Niño impacts, the intensity of the associated climatic perturbations differs among years, but the probable sequence of principal effects on *R*_eco_ is: first, a reduction in heterotrophic respiration in soil and woody tissue in combination with variable limitations on GPP; and, secondly, structural and physiological impacts developing following extended strong moisture limitation. The impact of any drought on NEP will vary where climatic and edaphic constraints on *R*_eco_ and GPP differ, but in general, the effects are very likely to oppose each other, dampening change in NEP rather than amplifying it, as previously modelled (e.g. Zeng *et al.* 2005). Where drought also leads to fire, then this large abiotic addition to *R*_eco_ could dominate the short-term carbon budget (van der Werf *et al.* 2004), rather than changes in NEP. Over decadal or longer time scales, continued drought can be expected lead to large emissions of CO_2_ from the land owing to substantial shifts in vegetation and soil properties. But in order to have confidence in longer-term predictions of rainforest–atmosphere interactions, we must first correctly understand and model short-term variations in the biophysical functioning of these ecosystems.

## Figures and Tables

**Figure 1:**
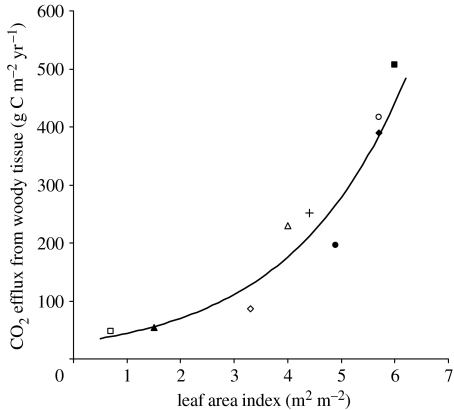
Variation with leaf area index (LAI) in CO_2_ efflux from above-ground woody tissue (*R*_wc_). Graph redrawn from Meir & Grace (2002), using new data for site 7 (*R*_wc_, Cavaleri *et al.* 2006; LAI, D. Clark 2007, personal communication) and data for two additional tropical rainforests, sites 8 and 9, near Manaus (C. Amazonia) and at Reserva Jaru (SW Amazonia), respectively (McWilliam *et al.* 1993; Kruijt *et al.* 1996; Meir 1996; Chambers *et al.* 2004). LAI was obtained directly for sites 1 and 5–8 and estimated indirectly for sites 2–4 and 9; the LAI for site 8 is taken from a nearby destructive harvest (McWilliam *et al.* 1993) because this estimate includes all tree sizes. *R*_wc_ was obtained from measurements of woody tissue CO_2_ efflux at all sites. Original $R_{\text{adj}}^{2}=0.85$, *p*=0.003, *n*=7 (Meir & Grace 2002); revised fit $R_{\text{adj}}^{2}=0.93$, *p*<0.0001, *n*=9 (*R*_wc_=29.1e^(0.45.LAI)^). TRF is tropical rainforest. Solid line, model; open square, site 1: Sahelian shrub, Niger; filled triangle, site 2: Ponderosa pine, USA; open diamond, site 3: Black spruce, Canada; plus sign, site 4: TRF, Cameroon; filled circle, site 5: deciduous broadleaf, USA; filled diamond, site 6: TRF, Brazil (Manaus); filled square, site 7: TRF, Costa Rica; open circle, site 8: TRF, Brazil (Manaus); open triangle, site 9: TRF, Brazil (Jaru).

**Figure 2:**
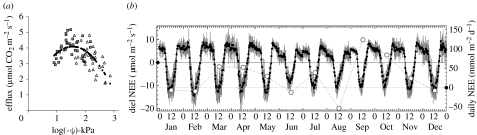
Moisture constraints on *R*_s_ and *R*_eco_. (*a*) The relationship between soil water potential and soil respiration (*R*_s_, μmol m^−2^ s^−1^; *R*^2^=0.43, *p*<0.001); data are from a soil drought experiment at an eastern Amazon rainforest, Caxiuana (Sotta *et al.* 2007). (*b*) Net ecosystem exchange (NEE) in a transitional forest, Sinop, Mato Grosso (Vourlitis *et al.* 2005). During the four-month dry season (May–August), *R*_eco_ is more sensitive to moisture stress than is maximum photosynthesis, *A*_max_ (a decline of 28% in *R*_eco_ versus 5% in *A*_max_; Vourlitis *et al.* 2005). Data show changes in diel and daily NEE of CO_2_. Positive values indicate CO_2_ release (mean NEE (±s.d.) of 30-min data (filled circle) and average daily NEE by month (open circles); horizontal solid line is diel NEE=0 and horizontal dashed line is daily NEE=0).

**Table 1:** Leaf dark respiration throughout the vertical profile of a rainforest at Caxiuana, in eastern Amazonia, following 4 years of artificial soil drought. Values represent the mean leaf dark respiration in μmol m^−2^ s^−1^±s.d. (*n*), at 25°C, and compare vegetation subjected to artificial soil drought and normal rainfall (Metcalfe *et al.* in preparation).

leaf height	control	drought
greater than 20 m	0.52±0.13 (14)	0.70 (1)
10–20 m	0.46±0.10 (14)	0.67±0.25 (12)
0–10 m	0.30±0.03 (5)	0.49±0.08 (15)
mean	0.46±0.15 (33)	0.57±0.19 (28)
